# NPY Neuron-Specific Y2 Receptors Regulate Adipose Tissue and Trabecular Bone but Not Cortical Bone Homeostasis in Mice

**DOI:** 10.1371/journal.pone.0011361

**Published:** 2010-06-29

**Authors:** Yan-Chuan Shi, Shu Lin, Iris P. L. Wong, Paul A. Baldock, Aygul Aljanova, Ronaldo F. Enriquez, Lesley Castillo, Natalie F. Mitchell, Ji-Ming Ye, Lei Zhang, Laurence Macia, Ernie Yulyaningsih, Amy D. Nguyen, Sabrina J. Riepler, Herbert Herzog, Amanda Sainsbury

**Affiliations:** 1 Neuroscience Research Program, Garvan Institute of Medical Research, Sydney, New South Wales, Australia; 2 Diabetes and Obesity Research Program, Garvan Institute of Medical Research, Sydney, New South Wales, Australia; 3 Faculty of Medicine, University of New South Wales, Sydney, New South Wales, Australia; 4 School of Medical Sciences, University of New South Wales, Sydney, New South Wales, Australia; The University of Queensland, Australia

## Abstract

**Background:**

Y2 receptor signalling is known to be important in neuropeptide Y (NPY)-mediated effects on energy homeostasis and bone physiology. Y2 receptors are located post-synaptically as well as acting as auto receptors on NPY-expressing neurons, and the different roles of these two populations of Y2 receptors in the regulation of energy homeostasis and body composition are unclear.

**Methodology/Principal Findings:**

We thus generated two conditional knockout mouse models, Y2^lox/lox^ and NPYCre/+;Y2^lox/lox^, in which Y2 receptors can be selectively ablated either in the hypothalamus or specifically in hypothalamic NPY-producing neurons of adult mice. Specific deletion of hypothalamic Y2 receptors increases food intake and body weight compared to controls. Importantly, specific ablation of hypothalamic Y2 receptors on NPY-containing neurons results in a significantly greater adiposity in female but not male mice, accompanied by increased hepatic triglyceride levels, decreased expression of liver cartinine palmitoyltransferase (CPT1) and increased expression of muscle phosphorylated acetyl-CoA carboxylase (ACC). While food intake, body weight, femur length, bone mineral content, density and cortical bone volume and thickness are not significantly altered, trabecular bone volume and number were significantly increased by hypothalamic Y2 deletion on NPY-expressing neurons. Interestingly, *in situ* hybridisation reveals increased NPY and decreased proopiomelanocortin (POMC) mRNA expression in the arcuate nucleus of mice with hypothalamus-specific deletion of Y2 receptors in NPY neurons, consistent with a negative feedback mechanism between NPY expression and Y2 receptors on NPY-ergic neurons.

**Conclusions/Significance:**

Taken together these data demonstrate the anti-obesogenic role of Y2 receptors in the brain, notably on NPY-ergic neurons, possibly via inhibition of NPY neurons and concomitant stimulation of POMC-expressing neurons in the arcuate nucleus of the hypothalamus, reducing lipogenic pathways in liver and/or skeletal muscle in females. These data also reveal as an anti-osteogenic effect of Y2 receptors on hypothalamic NPY-expressing neurons on trabecular but not on cortical bone.

## Introduction

Neuropeptide Y (NPY), a 36-amino acid peptide, is widely expressed in the central and peripheral nervous systems and is an important regulator of numerous physiological processes, including energy balance [1], [2]. In the central nervous system, NPY is present in regions such as the hypothalamus, cerebral cortex, brain stem, striatum and limbic structures [3], with the highest expression in the hypothalamic arcuate nucleus (Arc) [4]. Central control of energy balance is predominantly a function of hypothalamic pathways including key connections with the Arc. The Arc is located at the base of hypothalamus, and has direct access to circulating hormones due to a semi-permeable blood brain barrier [5]. Therefore, neurons within the Arc are able to respond rapidly to peripheral signals and then project broadly to other brain regions to modulate energy balance [6]. Located within the Arc are two major populations of neurons that are known to regulate energy balance: orexigenic NPY/agouti-related protein (AGRP) neurons and anorexigenic pro-opiomelanocortin (POMC)/cocaine and amphetamine-regulated transcript (CART) neurons [7]. Activation of NPY/AGRP neurons leads to release of NPY and AGRP, which stimulate appetite, fat storage and weight gain [8]. This occurs in association with effects of NPY on Y1 or Y5 receptors [9] and the ability of AGRP to antagonize melanocortin 3/4 (MC3/4) receptors in the paraventricular nucleus [8]. POMC/CART neurons produce alpha-melanocyte stimulating hormone (α-MSH), which acts to inhibit food intake, weight gain and energy balance by action on central MC3/4 receptors in the paraventricular nucleus [10]. In addition, NPY/AGRP neurons contact and inhibit adjacent POMC/CART neurons by releasing the inhibitory neurotransmitter gamma-aminobutyric acid (GABA) [5], [11]. NPY expression increases under conditions of food deprivation, which contributes to an increase in food intake and contributes subsequently to positive energy balance [12], [13], [14], [15]. Surprisingly, body weight and adiposity were normal in NPY-deficient mice fed with normal chow [16]. However, NPY deficiency does lead to attenuated responses to fasting and a high fat diet, and it also attenuates hyperphagia and the obesity syndrome of *ob/ob* mice [16], [17]. Consistent with an important role of NPY in the regulation of energy homeostasis, intracerebroventricular or hypothalamus-specific administration of NPY to normal rodents leads to defects characteristic of obesity, including hyperphagia, accelerated body weight gain, hyperleptinemia, hypercorticosteronemia, hyperinsulinemia and increased adiposity [18], [19], [20], [21]. More importantly, all of these neuroendocrine and metabolic effects of central NPY administration, notably increased adiposity, persist even when NPY-induced hyperphagia is prevented by pair-feeding, indicating that hyperphagia is not the only mechanism by which central NPY increases adiposity [18], [21].

NPY mediates its effects via a family of G protein-coupled receptors known as Y1, Y2, Y4, Y5, and y6 [4], each with varying distribution across central and peripheral tissues. Y2 receptors are widely expressed in areas of the brain such as the hypothalamus, hippocampus and brain stem [22], [23], and the involvement of Y2 receptors in energy homeostasis was initially supported by the demonstration that acute or chronic central administration of the Y2-preferring agonist NPY13-36 to normal rats deceases overnight feeding [24] and daily food intake [20]. Additionally, NPY13-36 has been shown to inhibit NPY release from rat hypothalamic sections *in vitro*, and this effect can be blocked by the selective Y2 receptor antagonist BIIE0246 [25]. More importantly, the gut-derived satiety peptide PYY3-36, released postprandially, reduces food intake in humans and in rodents via activation of Y2 receptors in the Arc [5]. Additional major support for the importance of Y2 receptors in the regulation of energy homeostasis comes from germline Y2 receptor knockout mice, which exhibit reduced adiposity [26]. Moreover, germline deletion of Y2 receptors leads to a significant reduction in adiposity or body weight and the type 2 diabetic syndrome of *ob/ob* mice, in the absence of reductions in food intake [27], [28]. Co-localization studies provided further evidence of the possible role of Y2 receptors in the hypothalamus – particularly within the Arc – in the regulation of energy homeostasis. Studies suggest that up to 80% of NPY-expressing neurons in the Arc are co-localized with Y2 receptors, and Y2 receptors have therefore been postulated to act as auto-receptors that modulate the synthesis and release of NPY [7], [28], in keeping with the effects of Y2 agonists on NPY release from hypothalamic explants *in vitro* [25]. However, there is no direct *in vivo* evidence that such ‘pre-synaptic’ Y2 receptors mediate effects on NPY expression and secretion and subsequently on energy homeostasis.

In addition to effects on energy regulation, lack of Y2 receptors, notably hypothalamic Y2 signalling as in conditional hypothalamus-specific Y2 receptor deletion in mice, results in increased cortical and trabecular (cancellous) bone mass [29], [30], [31], suggesting a potential link between the central control of energy homeostasis and bone homeostasis [32]. Site-specific overexpression of NPY in the hypothalamus of mice markedly reduced the capacity of osteoblasts to produce bone [29], [32]. Consistent with this effect being mediated by hypothalamic Y2 receptors, conditional deletion of Y2 receptors from the hypothalamus of adult mice produced a generalized increase in osteoblast mineral apposition rate, with no evidence of changes in the bone-resorption indices of osteoclast surface or osteoclast number [31]. Remarkably, mineral apposition rate in cancellous bone of hypothalamus-specific Y2^−/−^ mice was elevated 2-fold, in parallel with similar changes in cancellous bone volume [31].

Given the clear involvement of Y2 receptors in the regulation of energy homeostasis and bone mass, we investigated the effect of selective deletion of hypothalamic Y2 receptors in these processes, using a conditional Y2 receptor knockout mouse model (Y2^lox/lox^ mice) in which Y2 receptors were selectively deleted from the hypothalamus via adeno-associated viral Cre-recombinase delivery [26]. Furthermore, to explore the specific role of hypothalamic Y2 receptors on NPY neurons in the regulation of energy homeostasis and bone mass, we established a unique conditional NPY neuron-specific Y2 receptor deficient mouse model: NPYCre/+;Y2^lox/lox^, in which Y2 receptors were ablated only in hypothalamic neurons that express NPY via hypothalamic injection of doxycycline. Gene deletion in both models was induced in adult animals, thereby minimizing developmental influences.

## Materials and Methods

### Animals

Animal experiments were approved by the Garvan Institute/St Vincent's Hospital Animal Ethics Committee (Ethics No: HH #08/01) and were conducted in accordance with relevant guidelines and regulations. Mice were housed under conditions of controlled temperature (22°C) with a 12h light, 12 h dark cycle (lights on at 0700 h). Mice were fed a normal chow diet *ad libitum* (8% calories from fat, 21% calories from protein, 71% calories from carbohydrate, 2.6 kcal/g; Gordon's Speciality Stock Feeds, Yanderra, New South Wales, Australia). Water was available *ad libitum* for all mice. Generation of the conditional Y2^lox/lox^ and NPYCre mice was described previously [26], [32], [33].

### Generation of adult-onset hypothalamus-specific Y2 receptor knockout model (Y2^hyp^)

Region-specific Y2 receptor deletion was achieved in mice containing Y2 floxed alleles by injection with Cre-recombinase expressing recombinant adeno-associated viral (rAAV) vectors as previously described [26], [34]. In brief, 12-week-old Y2^lox/lox^ mice were anesthetized with a single dose of ketamine/xylazine (100/20 mg/kg, ip) (Mavlab, Slacks Creek, Queensland, Australia; Ilium Veterinary Products, Smithfiend, New South Wales, Australia) and placed on a Kopf stereotaxic frame (David Kopf Instruments, Tulunga, CA, USA). One microliter of rAAV vector containing either the Cre-recombinase gene or an empty cassette (1×10^14^ genomic copies/ml) was injected bilaterally into the hypothalamus at a rate of 0.1 µl/min using a 2-µl Hamilton syringe attached to syringe infusion pump (World Precision Instruments Inc, Walthan, MA, USA). The resultant mice were referred to as Y2^hyp^KO mice and Y2^lox/lox^mice, respectively. The injection coordinates relative to bregma were: anterioposterior −2.1 mm; mediolateral ±0.4 mm; dorsoventral −5.3 mm, corresponding to the arcuate nucleus (Arc) [35]. Animals were kept on a heating pad during surgery and until recovery.

### Generation of conditional NPY neuron-specific Y2 receptor knockout model

The inducible, conditional NPY neuron specific Y2 receptor knockout model (NPYCre/+;Y2^lox/lox^ mouse) was generated using a knock-in strategy. In brief, a mouse was generated in which the NPY gene was replace by a cassette containing the reverse tetracycline transactivator (rtTA) gene, and Cre-recombinase (Cre) under the control of a tetracycline responsive element (TRE). The construct was designed such that the endogenous NPY promoter drives expression of the rtTA gene, which in the absence of doxycycline is unable to bind and activate the TRE element to transcribe the Cre gene. It is only after the addition of doxycycline that Cre is expressed. The resultant homozygous conditional NPY-Cre-expressing transgenic mice on a mixed C57/BL6-129/SvJ background were crossed with Y2^lox/lox^ mice on the same background. Heterozygous offspring were crossed again to obtain heterozygous NPYCre/+;Y2^lox/lox^ mice. In the presence of the inducer doxycycline (Dox), Cre expression is induced, subsequently leading to excision of the loxP-flanked Y2 receptor gene and resulting in Y2 receptor ablation only in NPY-containing neurons. Adult-onset NPY neuron-specific Y2 receptor deletion was achieved by injection of 2 µg of doxycycline (Sigma-Aldrich Pty Ltd, Sydney, NSW, Australia) in 2 µL saline into the left lateral cerebral ventricle of 10–12 week-old NPYCre/+;Y2^lox/lox^ mice. The injection coordinates relative to bregma were: anterioposterior −0.34 mm; mediolateral ±1.0 mm; dorsoventral −2.5 mm, corresponding to the left lateral cerebral ventricle [35]. Surgery was carried out in the same manner as described in the previous section. Two groups of control mice were used for these experiments: NPYCre/+;Y2^lox/lox^ mice receiving central administration of saline, to control for possible effects of the knock-in construct and intracerebroventricular injection, and wild type mice treated with intracerebroventricular doxycycline, to control for possible effects of central doxycycline injection *per se*.

### DNA and RNA extraction and quantitative real-time PCR

Y2 receptor deletion was confirmed using genomic DNA extracted from the hypothalamic region of the brain as previously described [26]. In brief, genomic DNA was isolated from hypothalamic blocks from Dox- and saline-injected mice. PCR was performed with the following primers: Oligo A, 5′-AGCATCCAGAGAAGTGCAAC-3′ and Oligo B, 5′-TTAACATCAGCTGGCCTAGC-3′. This primer pair only produces a PCR product, 250 base pairs long, from DNA in which the Y2-receptor gene has been deleted. PCR conditions were 5 minutes denaturation at 95°C followed by 35 cycles of 1 minute at 95°C, 1 minute at 61°C and 40 seconds at 72°C. Total RNA from the hypothalamic region of NPYCre/+;Y2^lox/lox^ and NPYCre/+;Y2^−/−^ mice was isolated using Trizol® Reagent (Sigma, St Louis, MO, USA) following the manufacturer's protocol. The quality and concentration of total RNA was measured by a spectrophotometer (Nanodrop 1000, NanoDrop Technologies, LLC, USA). One microgram (1 µg) of total RNA was reverse transcribed into cDNA using the Superscript III First-Strand Synthesis System (Invitrogen, Mount, Waverley, VIC, Australia). Quantitative real-time PCR using primers for the Y2 receptor gene was carried out on a LightCycler® (LightCycler® 480, Roche Applied Science, Germany) using the SensiMix™ Probe (Bioline Australia Pty Ltd, Alexandria, NSW, Australia) following the manufacturer's instructions. Expression of the housekeeping gene, ribosomal protein L13A (RPL-13A), was carried out in the same manner and was used to normalize expression level of the Y2 receptor.

### Determination of food intake and body weight

Mice were housed individually and body weight was measured twice a week at the same time of day. At 15 weeks of age, spontaneous daily food intake was measured over 3 consecutive days in individually housed mice. Actual food intake was calculated as the weight of pellets taken from the food hopper minus the weight of food spilled in the cage. The weight of spilled food per day was determined as the 24-h increase in weight of the cage bedding, after removing all feces and air-drying to eliminate weight changes due to urine and water bottle drips. The average was used for statistical analysis. At 16 weeks of age, fasting-induced food intake was carried out after fasting for 24 h at 900 h, then determining food intake as described above at 2, 4, 8, 24, 48 and 72 hours after re-introduction of food. Body weight was tracked at the same time each day before and up to 72 hours after the 24-hour fast.

### Rectal temperature measurements

At 14–15 weeks of age, body temperature was measured at 8:30–9:00 h with a rectal thermometer (Physitemp Instruments Inc, Clifton, NJ, USA). Temperature readings were taken within 10 seconds of removing the mouse from its cage. Repeat readings were taken from each mouse on 3 consecutive days, and the average of the three readings was used for statistical analysis.

### *In situ* hybridization and densitometry

Mice were culled as described below and 20 µm thick coronal sections of fresh frozen brains were cut and thaw-mounted on charged slides and stored at −20°C until use. For radioactive *in situ* hybridisation, DNA oligonucleotides complementary to mouse NPY (5′-GAGGGTCAGTCCACACAGCCCCATTCGCTTGTTACCTAGCAT-3′) and POMC (5′-TGGCTGCTCTCCAGGCACCAGCTCCACACATCTATGGAGG-3′) were labelled with [^35^S] thio-dATP (Amersham Biosciences, Little Chalfont, Buckinghamshire, UK) using terminal deoxynucleotidyltransferase (Roche, Mannheim, Germany). The expression levels of NPY and POMC mRNA were evaluated by measuring silver grain densities over individual neurons from photo-emulsion-dipped sections as described previously [26]. Background labeling was uniform and never exceeded 5% of specific signal.

### Glucose tolerance test (GTT)

At 17 weeks of age, mice were fasted for 16 h before intraperitoneal injection of a 10% D-glucose solution (1.0 g/kg). Blood samples were obtained from the tail tip at the indicated times, and glucose levels were measured using a glucometer (AccuCheck II; Roche, New South Wales, Castle Hill, Australia).

### Indirect calorimetry and determination of physical activity

Oxygen consumption rate (*V*o_2_) and carbon dioxide output (*V*_CO2_) were measured using an open circuit eight-chamber indirect calorimeter (Oxymax series; Columbus Instruments, Columbus, OH, USA) with airflow of 0.6 L/min. Studies were commenced after 24 h of acclimation to the metabolic chamber (dimensions 20×10×12.5 cm). *V*o_2_ and *V*_CO2_ were measured in individual mice at 27-min intervals over a 24-h period under a consistent environmental temperature of 22°C. The respiratory exchange ratio (RER) was calculated as the quotient of *V*_CO2_/*V*o_2_, with the value of 1 representing pure carbohydrate oxidation and the value of 0.7 representing pure fat oxidation [36], [37]. Energy expenditure was calculated as calorific value (CV) x *V*o_2_, where CV is 3.815+1.232 x RER [38]. During the calorimetry study, mice had *ad libitum* access to food and water.

During calorimetry, relative physical activity over 24 h in mice was also measured using a monitoring system from Columbus Instruments that reports activity as sequential beam breaks on an infrared grid. Cumulative ambulatory counts of X and Y directions were summed for 1 h intervals.

### Bone densitometry and body composition analysis and microcomputed tomography (micro-CT)

Whole body bone mineral density (BMD), bone mineral content (BMC) and fat and lean body mass were measured on mice ventral side down at 15 weeks of age using dual-energy X-ray absorptiometry (DXA; Lunar PIXImus2 mouse densitometer, GE Medical Systems, Madison, WI, USA) as stipulated by the manufacturer. Mice were anesthetized with isoflurane, scanned, and BMD, BMC and total body fat and lean body mass were determined using the program provided by the manufacturer. The head and the tail of the animal were excluded from the analysis of body composition. Whole femoral BMD and BMC were measured in excised left femora. Femora were DXA scanned with tibiae attached and the knee joint in flexion to ninety degrees to ensure consistent placement and scan of the sagittal profile. Following fixation, left femora were cleaned of muscle and analysed using micro computed tomography (micro-CT) with a Skyscan 1174 scanner and associated analysis software (Skyscan, Aartselaar, Belgium). During scanning, the femora were enclosed in a rigid plastic tube filled with 70% ethanol and prevented from moving by polystyrene packing at the proximal end. The X-ray source was set at 50 kV and 800 µA. Scanning was carried out with a 0.5 mm aluminum filter in place in order to reduce noise, and sharpening was set to 40%. Image projections were acquired over an angular range of 180° (angular step of 0.4°) with pixel size of 6.2 µm and the exposure set to 3600 ms. The image slices were reconstructed using NRecon (Skyscan's volumetric reconstruction software), which uses a modified Feldkamp algorithm. Reconstruction was carried out with automated misalignment compensation for each individual sample and the following settings for the whole study samples: beam-hardening correction set to 30%, ring artifact correction set to 5, smoothing set to 4, and the threshold for defect pixel masking set to 10%. The reconstructed images were then straightened using Dataviewer software (Skyscan). Trabecular bone of the distal femur was selected for analysis by fitting a 1 mm×1.5 mm elliptic region of interest in the center of the femur, starting at 40 slices (0.25 mm) proximally from the growth plate and extending a further 160 slices (0.99 mm) in the proximal direction. Cortical bone was analysed in 150 slices (0.93 mm) selected at 730 slices (4.53 mm) proximal from the growth plate. Thresholding was applied to the images to segment the bone from the background and the same threshold setting was used for all the samples. The following 3-dimensional parameters were calculated: trabecular bone volume as a percent of tissue volume, trabecular number, trabecular thickness, cortical bone volume and cortical bone thickness.

### Western blotting

Liver and muscle samples taken from animals culled as described below were homogenized in RIPA buffer (25 mM Tris•HCl pH 7.6, 150 mM NaCl, 1% NP-40, 1% sodium deoxycholate, 0.1% SDS) supplemented with Complete Protease Inhibitor Cocktail Tablets (Complete Mini, Roche Diagnostic, Mannheim, Germany). After centrifugation, clear lysates were collected and protein concentrations were measured using microplate spectrophotometer (Spectramax Plus^384^, Molecular Devices Inc., Silicon Valley, CA, USA) using a reagent from Biorad (Biorad, Gladesville, NSW, Australia). Equal amounts of tissue lysates (20 µg protein) were resolved by SDS-PAGE and immunoblotted with antibodies against liver and muscle carnitine palmitoyltransferase (CPT-1) (Santa Cruz Biotechnology, Santa Cruz, CA, USA), phosphorylated acetyl-CoA carboxylase (p-ACC), fatty acid synthase (FAS) (Cell Signaling, Beverly, MA, USA) and peroxisome proliferator-activated receptor (PPAR) γ coactivator (PGC)-1α (Calbiochem, Merck Pty Ltd Kilsyth, VIC, Australia). Immunolabelled bands were quantified by densitometry.

### Tissue collection, serum hormone analysis and assay of tissue triglyceride content

At the completion of each study, mice were culled by cervical dislocation between 12:00–15:00 h, trunk blood was collected, allowed to clot at room temperature, centrifuged, and resultant sera were stored at −20°C for subsequent analysis as described below. Brains were removed and immediately frozen on dry ice. White adipose tissue (WAT) depots (right inguinal, right retroperitoneal, reproductive (right ovarian or right epididymal for female and male mice, respectively), and mesenteric), brown adipose tissue (BAT), pancreas, liver, skeletal muscle (deeper layer of quadriceps containing mostly red fibres), right kidney, heart and right testis were collected, weighed and stored at −80°C for further analysis. The weight of WAT depots were summed together and expressed as total WAT weight, normalized as a percentage of body weight.

Serum insulin levels were measured using a radioimmunoassay kit (Linco Research, St Louis, MO, USA). Serum concentrations of glucose, triglycerides and free fatty acid were determined with colorimetric kits (Trace Scientific, Melbourne, Australia and Sigma Diagnostic, St. Louis, MO). Serum, muscle and liver triglyceride contents were determined using a colorimetric assay kit (Triglycerides GPO-PAP; Roche Diagnostics, Indianapolis, IN, USA) as previously described [39].

### Statistical analysis

Results were assessed by factorial ANOVA. When there was a significant overall effect or an interaction effect, Bonferroni post-hoc tests were performed to identify differences using GraphPad Prism 5 (Version 5.0a, GraphPad Software, Inc). For all statistical analysis, a p-value <0.05 was considered to be statistically significant.

## Results

### Effect of hypothalamus-specific Y2 receptor deletion on body weight, food intake and adiposity

**Figure 1:**
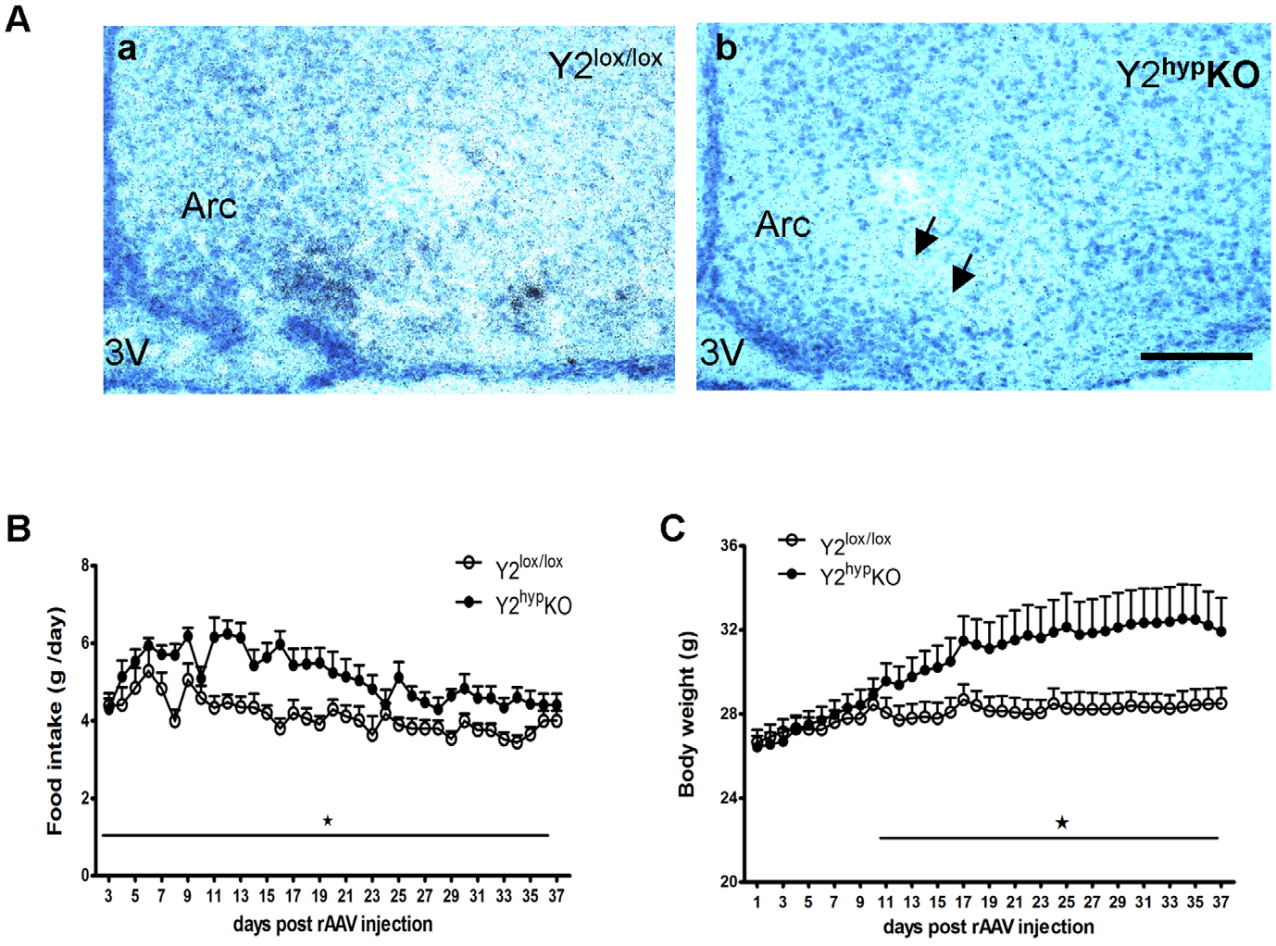
Hypothalamus-specific Y2 deletion increases food intake and body weight. (A) Marked reduction in Y2 receptor mRNA in the Arc of male Y2^lox/lox^ mice – as detected by *in situ* hybridization – after injection of Cre-expressing adenovirus into the hypothalamus (Y2^hyp^ KO mice, Figure A (b)) compared to that in male Y2^lox/lox^ mice (Figure A (a), scale bar 40 µm). Food intake (B) and body weight (C) in male Y2^hyp^KO mice compared to that of Y2^lox/lox^-empty vector-injected control mice. Values are means ± SEM of 8 male mice per group. * p<0.05 versus Y2^lox/lox^ control mice. Arc: Arcuate nucleus of the hypothalamus; 3V: third cerebral ventricle.

In order to evaluate the role of hypothalamic Y2 receptors in the regulation of energy balance, Y2 receptors were selectively deleted from the hypothalamus by injection of a recombinant adeno-associated viral vector expressing Cre-recombinase (rAAV-Cre) into the hypothalamus of adult male Y2^lox/lox^ mice. The deletion of hypothalamic Y2 receptor deletion was confirmed by *in situ* hybridisation (Figure 1A). Compared to empty vector-injected control mice (Figure 1A(a)), Y2 mRNA expression is significantly reduced in the Arc of rAAV-Cre-injected Y2^hyp^KO mice (Figure 1A(b)). Compared to Y2^lox/lox^ control mice, 24-hour spontaneous food intake of Y2^hyp^KO mice was significantly increased (Figure 1B). The two groups of mice were of similar initial body weight, with no significant difference in body weight in the first 13 days after rAAV injection (Figure 1C). However, from day 13 onwards, Y2^hyp^KO mice gained significantly more weight compared to controls (Figure 1C). Increased body weight and food intake were associated with a trend towards increased mass of dissected white adipose tissue depots (expressed as a percent of body weight) upon completion of the study at 38 days after rAAV injection. Indeed, the summed weight of white adipose tissue depots was 1.68±0.13 versus 2.09±0.40 percent of body weight in Y2^lox/lox^ control and Y2^hyp^KO mice, respectively (data are mean ± SEM of 8 mice per group; p = 0.15). There was no statistically significant difference in relative weights of brown adipose tissue depots between the two groups (0.227±0.013 versus 0.215±0.02 percent of body weight in Y2^lox/lox^ and Y2^hyp^KO mice, respectively. Data are mean ± SEM of 8 mice per group). The other organ weights as a percent of body weight in Y2^hyp^KO mice did not differ significantly from those in control mice (data not shown). These data suggests that lack of hypothalamic Y2 receptors leads to positive energy balance characterized by hyperphagia and increased body weight gain, which is consistent with the known role of Y2 receptors as a mediator of PYY3-36-induced satiety [5]. Another possible contributor to weight gain in this model is the increased bone mass that has been reported in response to hypothalamus-specific Y2 receptor deletion in mice [31]. However, as total body bone mineral content represents less than 2% of body weight, increases in bone mass are unlikely to contribute significantly to an increase in overall body weight.

### Generation of a conditional NPY neuron-specific Y2 receptor knockout model

**Figure 2:**
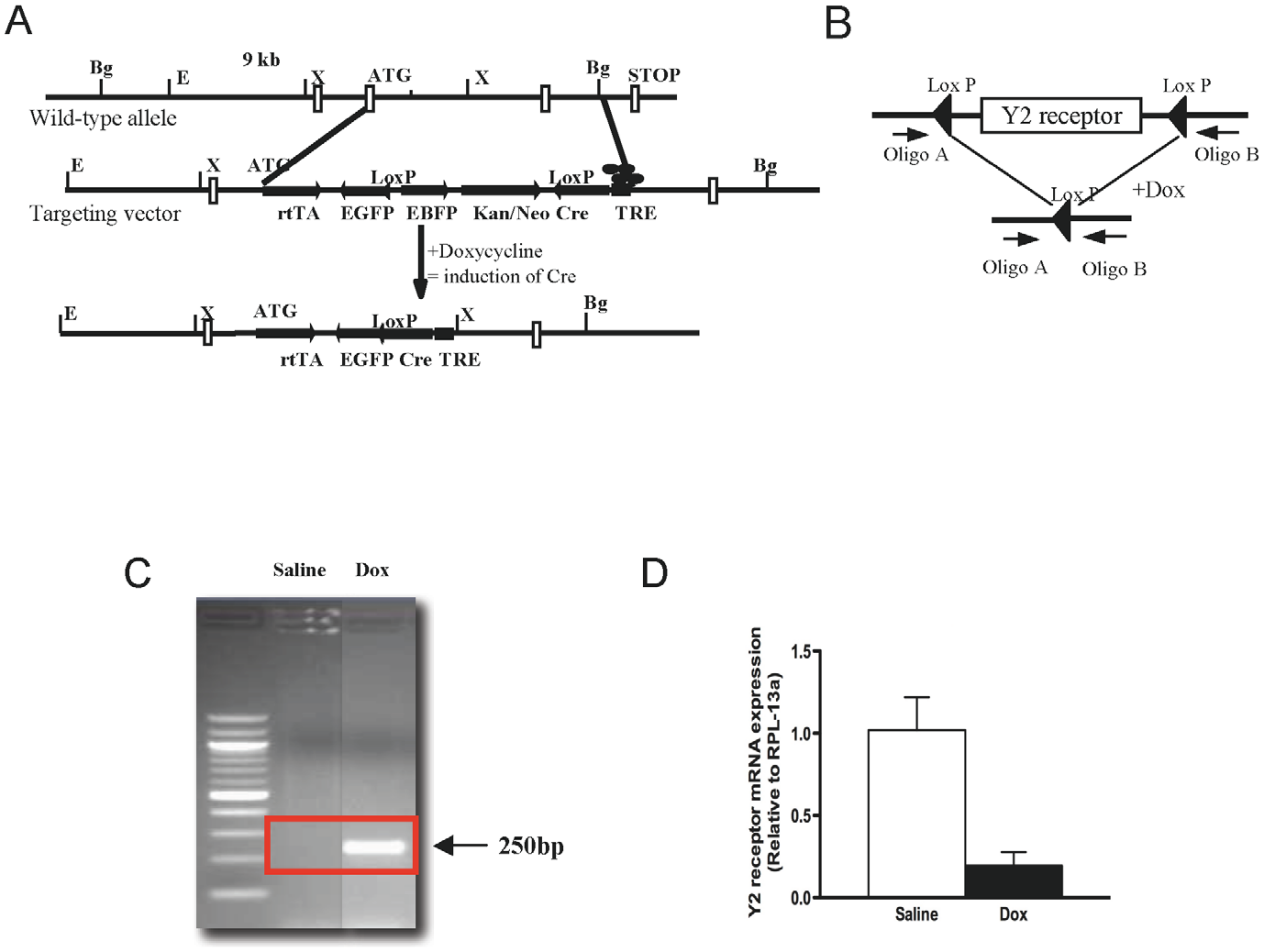
Schematic diagram of NPY-Cre-GFP knock-in strategy and confirmation of Y2 receptor deletion. (A) Conditional NPY neuron-specific Y2 receptor knockout mice (NPYCre/+;Y2^lox/lox^ mice) were generated using a knock-in strategy. The inducible NPY neuron-specific expression of the Cre gene is achieved by fusion of the initiation codon of the NPY gene with that of the tetR gene, enabling the endogenous NPY promoter to drive the expression of this regulatory protein upon addition of doxycycline (Dox). (B) Schematic drawing of position of oligonucleotide primers (Oligo A & B) used for PCR verification of Y2 receptor knockout. (C) Using genomic DNA extracted from the hypothalamus of NPYCre/+;Y2^lox/lox^ mice, Oligos A and B produced a 250 base pair (bp) product from a Dox-injected mouse, demonstrating deletion of the Y2 receptor gene, but no product was produced using DNA from a saline-injected control mouse. (D) Y2 receptor mRNA extracted from the hypothalamus of male NPYCre/+;Y2^−/−^ mice was significantly reduced compared to that of saline-injected mice.

The majority of hypothalamic Y2 receptors are expressed on NPY-containing neurons [28]. Therefore, in order to examine whether the observed anabolic effect of hypothalamic Y2 receptor deletion was due to the absence of Y2 receptors on these neurons, a conditional knockout mouse in which Y2 receptors were deleted specifically from NPY-expressing neurons was generated using a knock-in strategy which allows for an inducible approach. Firstly, an NPY-Cre ‘knock in’ targeting vector was constructed (Figure 2A), in which the coding exons of the NPY gene were replaced with a cassette consisting of the tetracycline repressor (tetR) gene, followed by an enhanced green fluorescent protein (EGFP) gene in the opposite orientation, a neo cassette for selection and a Cre gene under the control of the tetracycline responsive element (TRE), also in the opposite orientation. The stop codon of the Cre gene and the start codon of the EGFP gene were modified to contain loxP sites in the same orientation, allowing for the generation of a Cre-EGFP fusion product for colour detection after Cre-mediated excision of the intervening sequence. The EGFP gene, missing a functional ATG, is unable to produce a protein on its own. The inducible NPY neuron-specific expression of the Cre gene is achieved by the fusion of the initiation codon of the NPY gene with that of the tetR gene, thus enabling the endogenous NPY promoter to drive the expression of this regulatory protein (Figure 2A). The tetR gene product on its own is inactive, and it is only after the addition of doxycycline (Dox) that it gains activity as a transcription factor by binding and activating the TRE promoter region and transcribing the adjoining gene, in this case Cre [40]. Mice containing this knock-in construct were generated using standard ES technology, and the resultant mice were bred to homozygosity. Homozygous, inducible NPY-driven Cre-EGFP-knock-in mice were then crossed with homozygous Y2^lox/lox^ mice to generate heterozygous NPYCre/+;Y2^lox/lox^ mice for use in the present study.

### NPY neuron-specific Y2 receptor gene deletion

To examine the specific role of Y2 receptors on NPY-expressing neurons in the brain, particularly in the hypothalamus, in the regulation of energy balance, 12 week-old conditional NPYCre/+;Y2^lox/lox^ mice were injected with Dox into the lateral cerebral ventricle to induce selective deletion of Y2 receptors in these neurons. To confirm deletion of the Y2 receptor gene, genomic DNA was isolated from the hypothalamus and subjected to PCR analysis. Primers that are only able to produce a 250 base pair (bp) product once the Y2-receptor gene has been removed were used (Figure 2B, 2C). No product was produced using DNA isolated from the hypothalamus of saline-injected control mice, but the injection of Dox directly into the brain resulted in successful deletion of Y2 receptors (Figure 2C). Additional confirmation of successful gene deletion was obtained by quantitative real-time PCR showing a significant 90% reduction in hypothalamic Y2 receptor mRNA expression in Dox-injected NPYCre/+;Y2^−/−^ mice, as shown in Figure 2D. There was no induction of Cre expression in non-brain tissues, as determined by PCR in the liver, kidney, muscle and white adipose tissue (data not shown).

### Altered expression of neuropeptides in the hypothalamus of conditional NPY neuron-specific Y2 null mice

**Figure 3:**
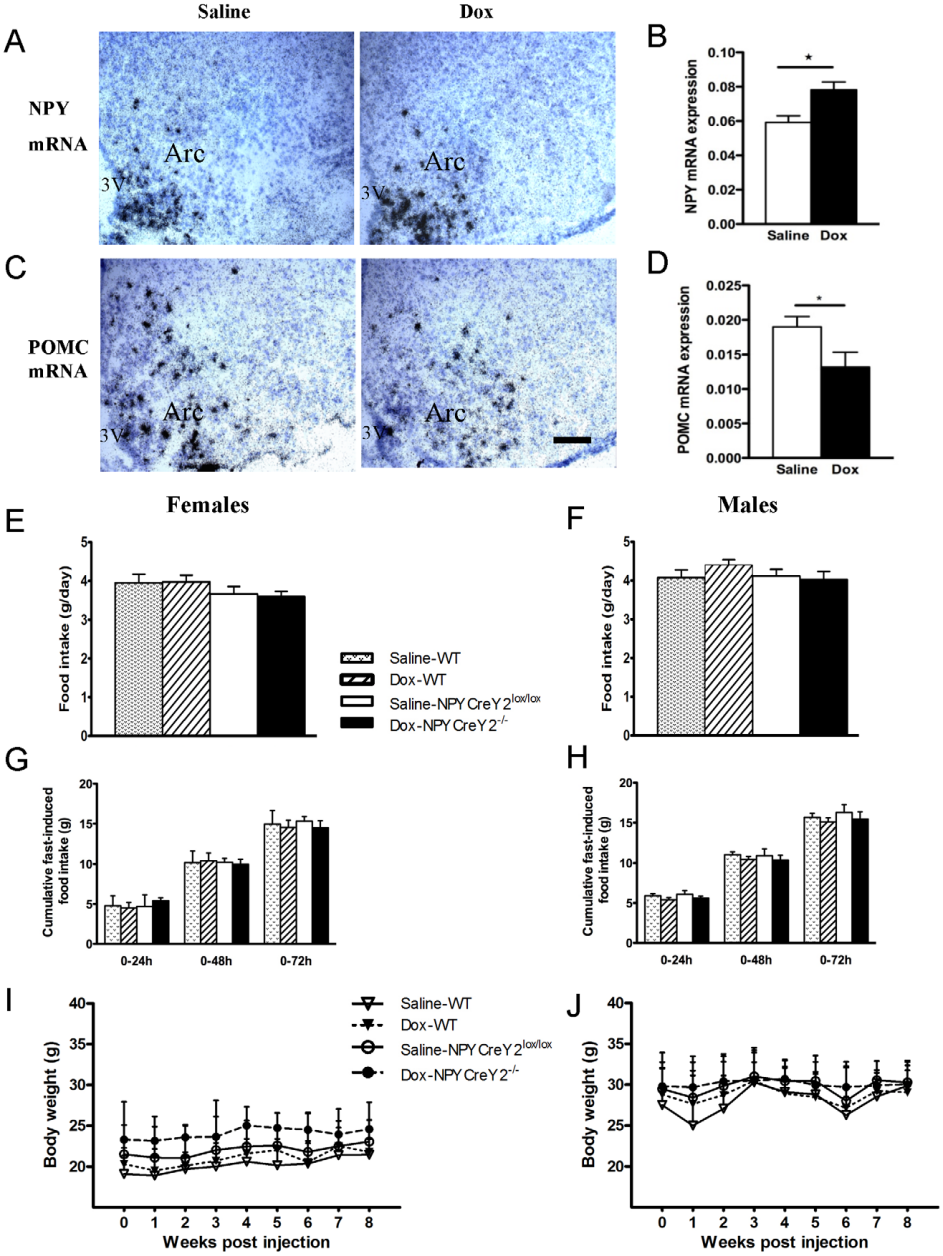
NPY neuron-specific Y2 deletion in the hypothalamus increases NPY and decreases POMC expression in the arcuate nucleus with no significant effects on body weight, spontaneous or fasting-induced food intake. (A, C) Bright-field photomicrographs of coronal brain sections, including the Arc, obtained from saline-injected control and doxycycline (Dox)-injected male NPYCre/+;Y2^−/−^ mice after *in situ* hybridization for NPY and proopiomelanocortin (POMC) mRNA. Scale bar, 40 µm. (B, D) Quantification of mean labeling intensity of neurons from *in situ* hybridization, given as percentage coverage of neuronal surface by silvergrains ± SEM of 5 male mice per group. *: p<0.05 versus saline-injected control mice. Arc: Arcuate nucleus of the hypothalamus; 3V: third cerebral ventricle. Spontaneous food intake (E, F), cumulative fasting-induced food intake (G, H) and body weight (I, J) are not altered by NPY neuron-specific Y2 receptor deletion in the hypothalamus. Data are mean ± SEM of 5 or more mice per group.

The localisation of Y2 receptors on NPY-expressing neurons suggests a potential role for Y2 as an auto-receptor regulating the expression and release of NPY and other co-localized neurotransmitters such as AGRP [25]. In order to explore the effect of NPY-neuron-specific Y2 receptor knockout on hypothalamic neuropeptide expression, we investigated the mRNA levels of two important neurotransmitters in the Arc by *in situ* hybridization. Consistent with its reported role as an auto-receptor, the absence of Y2 receptors on NPY-containing neurons leads to a significant increase in NPY mRNA expression in the Arc, as seen in Dox-injected mice compared to saline-injected control mice (Figure 3A, 3B). Interestingly, mRNA expression for proopiomelanocortin (POMC), the precursor for the anorexigenic alpha melanocyte stimulating hormone (α-MSH), expressed in neurons interacting with NPY-ergic neurons in the Arc, is significantly down-regulated in Dox-injected NPYCre/+;Y2^lox/lox^ mice compared to that in saline-injected control mice (Figure 3C, 3D). This finding suggests that altered NPY neuron function influences neighbouring POMC neurons in the Arc. These results further confirm successful deletion of Y2 receptors in our model, and more importantly they demonstrate that deletion of Y2 receptors on NPY-ergic neurons of adult mice has significant effects on hypothalamic NPY and POMC expression, highlighting the functional existence of a negative feedback mechanism between NPY and its Y2 auto-receptors.

### Effect of conditional NPY neuron-specific Y2 receptor deletion on food intake and body weight

Increased NPY and decreased POMC mRNA expression in the hypothalamus suggested that deletion of Y2 receptors on NPY-ergic neurons could alter food intake. Interestingly, in contrast to the knockout mice in which Y2 receptors were selectively deleted from the hypothalamus (Figure 1), 24-hour spontaneous food intake (Figure 3E, 3F), food intake in response to 24-hour fasting (Figure 3G, 3H), daily water intake and faecal output (Table 1) were not different in NPYCre/+;Y2^lox/lox^ mice injected with doxycycline (NPYCre/+;Y2^−/−^) of either gender compared to wild type controls injected with saline or doxycycline, or NPYCre/+;Y2^lox/lox^ mice injected with saline. All groups of mice recovered lost body weight in an equivalent manner after 24-hour fasting (data not shown). Additionally, body weight gain monitored over an 8-week period after intracerebroventricular injection was not different in Dox-injected NPYCre/+;Y2^lox/lox^ mice versus saline-injected NPYCre/+;Y2^lox/lox^ or wild type mice of either gender (Figure 3I, 3J). Nasal-anal length was also not significantly different between NPYCre/+;Y2^−/−^ mice and NPYCre/+;Y2^lox/lox^ or wild type control mice (data not shown).

**Table 1:** Effect of deleting Y2 receptors from hypothalamic NPY-expressing neurons on body weight, feeding parameters, body composition and glucose metabolism.

Parameters	Female				Male			
	Wild type		NPYCre/+;Y2^lox/lox^		Wild type		NPYCre/+;Y2^lox/lox^	
	Saline (5)	Dox(5)	Saline (7)	Dox(8)	Saline (5)	Dox(7)	Saline (6)	Dox(12)
**Daily water intake (g)**	4.69±0.34	5.34±0.31	5.33±0.19	4.62±0.46	5.44±0.64	5.36±0.55	4.54±0.25	4.43±0.19
**Daily fecal output (g)**	1.30±0.12	1.2±0.06	1.07±0.11	1.16±0.13	1.31±0.12	1.36±0.10	1.36±0.07	1.35±0.10
**Fasting serum glucose (um/L)**	6.53±0.74	6.55±0.85	7.90±0.80	6.30±1.50*	9.60±0.35	9.01±0.16	5.76±0.62	6.06±0.67
**Glucose area under curve (mmol/l/120 min)**	1060±43	1085±108	1080±36	906.8±108	1328±91	1247±32	1073±188	1067±58

Values are means ± SEM of 5–12 wild type or NPYCre/+;Y2^lox/lox^ mice injected into the hypothalamus with either saline or doxycyline (Dox), as indicated in parentheses.

*p<0.05 versus sex-matched NPYCre/+;Y2^lox/lox^ mice.

Despite no changes in food intake, body weight or length measured in the above studies, when male NPYCre/+;Y2^−/−^ mice were analysed over a 2-day period in indirect calorimetry cages, a significant increase in body weight was observed in these mice compared to age-matched wild type and saline-injected NPYCre/+;Y2^lox/lox^ control mice, which actually displayed a decrease in body weight during this change in housing (Table 2). While Dox injection *per se* had no significant effect on most of the parameters investigated in this study, Dox-induced deletion of Y2 receptors on central NPY-ergic neurons in male mice is associated with a trend to increased spontaneous food intake and a significant decrease in rectal temperature, with no change in faecal output (Table 2). This transient positive energy balance in male NPYCre/+;Y2^-/-^ mice relative to controls occurs without any changes in oxygen consumption, respiratory exchange ratio (RER), an index of metabolic fuel selection, or physical activity (Table 2). Similarly to males, female NPYCre/+;Y2^−/−^ mice gained significantly more weight compared to wild type or saline-injected NPYCre/+;Y2^lox/lox^ control mice while in the indirect calorimetry chambers (Table 2). In contrast to males, this transient positive weight gain is accompanied by unaltered daily food intake, fecal output or rectal temperature, but a significant increase in oxygen consumption, notably in the light phase, with – as in male mice – no change in RER (Table 2). Compared to female wild type or saline-injected NPYCre/+;Y2^lox/lox^ control mice, female NPYCre/+;Y2^−/−^ mice exhibited a significantly lower level of physical activity (Table 2). Taken together, these observations demonstrate that Y2 receptors on NPY-ergic neurons are not involved in the regulation of body weight or feeding behaviour under basal conditions or after food deprivation, but they may be important regulators of aspects of energy homeostasis controlling body weight under conditions such as stress.

**Table 2:** Effect of deleting Y2 receptors from hypothalamic NPY-expressing neurons on body weight change, feeding parameters, rectal temperature, oxygen consumption (VO2), respiratory exchange ratio (RER) and physical activity as determined during indirect calorimetry.

Parameters	Female				Male			
	WT		NPYCre/+;Y2^lox/lox^		WT		NPYCre/+;Y2^lox/lox^	
	Saline (5)	Dox (5)	Saline (7)	Dox (8)	Saline (5)	Dox (7)	Saline (6)	Dox (12)
**Body weight change (% initial BW)**	0.65±0.86	−0.65±0.79 #	0.74±0.27	2.10±0.82 *	−0.64±0.46	−0.28±0.33	−1.18±0.61	1.193±0.35 *
**Daily food intake (g)**	4.52±0.30	4.57±0.05	4.42±0.05	4.56±0.16	4.74±0.49	4.29±0.10	4.77±0.18	5.04±0.22
**Daily fecal output (g)**	0.80±0.07	0.75±0.09	1.38±0.32	0.98±0.13	0.96±0.08	0.93±0.06	1.11±0.12	1.22±0.08
**Rectal temperature (C)**	37.57±0.10	36.78±0.17	36.70±0.06	36.67±0.13	36.84±0.10	36.93±0.14	36.77±0.01	36.16±0.02 *
**VO2 (kcal/kg/hr)**								
**24 hour**	4635.65±117.24	4480.13±106.17	3855.99±135.88	4148.815±147.0*	3680.47±90.10	3665.75±54.22	3385.74±81.37	3616.89±74.79
**Light phase**	4349.00±81.34	4086.16±98.36	3503.74±186.44	3879.31±157.69*	3400.00±77.19	3370.87±35.75	3135.42±105.78	3338.28±72.80
**Dark phase**	4921.84±157.37	4874.10±148.61	4208.25±127.88	4418.32±150.05	3960.58±113.78	3960.65±84.227	3636.06±98.75	3895.50±93.06
**RER**								
**24 hour**	0.987±0.020	0.999±0.023	0.954±0.019	0.983±0.018	0.943±0.008	0.957±0.006	0.965±0.018	0.974±0.009
**Light phase**	0.950±0.021	0.973±0.027	0.918±0.021	0.947±0.026	0.893±0.012	0.922±0.010	0.930±0.023	0.961±0.015
**Dark phase**	1.023±0.020	1.026±0.027	0.990±0.022	1.019±0.011	0.992±0.01	0.991±0.008	1.000±0.017	0.961±0.009
**Physical activ ity (ambulatory counts/hr)**								
**24 hour**	592.8±138.2	738.8±147.7	227.3±62.1	161.6±28.8 *	311.7±36.1	325.0±30.7	149.2±15.6	185.3±27.9
**Light phase**	241.8±58.0	253.9±36.7	62.6±16.8	55.8±10.7	128.4±25.4	106.7±19.2	54.74±7.18	81.3±12.4
**Dark phase**	943.8±219.0	1223.8±291.7	384.7±111.3	267.4±49.5	494.9±59.1	543.3±49.9	243.6±24.8	289.4±48.0

Values are means ± SEM of 5–12 wild type or NPYCre/+;Y2^lox/lox^ mice injected into the hypothalamus with either saline or doxycyline (Dox). The number of mice in each group is indicated in parentheses.

# p<0.05 versus sex-matched saline-injected WT mice.

*p<0.05 versus sex-matched saline-injected NPYCre/+;Y2^lox/lox^ mice. Body weight change is expressed as a percentage of initial body weight after 2 days in the indirect calorimetry chambers.

### Sex-specific changes in body composition in NPY neuron specific Y2 receptor null mice

**Figure 4:**
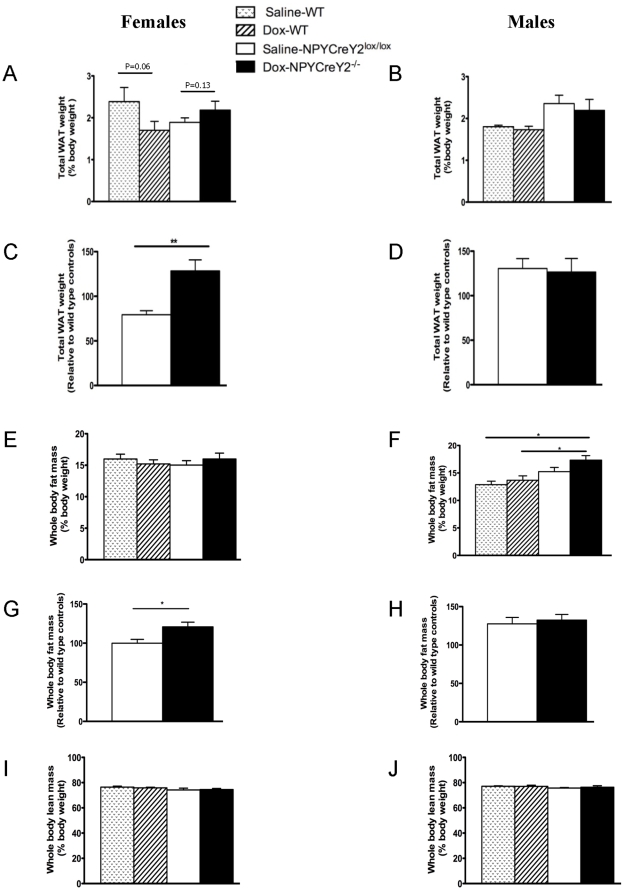
Effects of deleting Y2 receptors from hypothalamic NPY-expressing neurons on body composition in female and male mice. (A, B) Total weight of dissected white adipose tissue (WAT) depots, expressed as a percent of body weight, in wild type or NPYCre/+;Y2^lox/lox^ mice injected into the hypothalamus with either saline or doxycyline (Dox). (C, D) Total WAT weight of Dox-injected NPYCre/+;Y2^lox/lox^ mice relative to that of Dox-injected wild type mice (black columns) versus saline-injected NPYCre/+;Y2^lox/lox^ mice relative to saline-injected wild type mice (open column). (E, F) whole body fat mass (expressed as a percent of body weight), (G, H) whole body fat mass of Dox-injected NPYCre/+;Y2^lox/lox^ mice relative to that of Dox-injected wild type mice (black columns) versus saline-injected NPYCre/+;Y2^lox/lox^ mice relative to saline-injected wild type mice (open column). (I, J) whole body lean mass (expressed as a percent of body weight) of wild type or NPYCre/+;Y2^lox/lox^ mice injected into the hypothalamus with either saline or doxycyline, as determined by dual energy X-ray absorptiometry (DXA). Data are mean ± SEM of 5 or more mice per group. *: p<0.05 for the comparison indicated by horizontal bar.

To investigate the possibility that Y2 receptors on NPY producing neurons influence body composition, Dox-induced NPYCre/+;Y2^−/−^ mice were compared with age-matched saline-injected NPYCre/+;Y2^lox/lox^ control mice and saline- or Dox-injected wild type mice. Female but not male wild type mice injected with Dox showed a trend to reduced total fat mass (expressed as a percent of body weight) (p = 0.06) (Figure 4A, 4B). However, compared to saline-injected NPYCre/+;Y2^lox/lox^ control mice, female but not male NPYCre/+;Y2^−/−^ knockout mice display a trend towards increased percent adiposity after Dox injection (Figure 4A, 4B), suggesting that NPY neuron-specific Y2 receptor ablation increases relative fat mass in female mice. Two-way ANOVA revealed a significant interaction effect between genotype and treatment, indicating that genotype modified the outcome of Dox treatment. To examine the effect of Y2 receptor deletion *per se*, independent of the effect of Dox, the weights of dissected WAT depots (expressed as a percent of body weight) in NPYCre/+;Y2^−/−^ mice were normalised to corresponding values of Dox-injected wild type mice, and the results were compared to that of saline-injected NPYCre/+;Y2^lox/lox^ mice relative to saline-injected wild type mice. Thus, an anabolic effect of NPY neuron-specific Y2 receptor deletion became apparent: summed fat weights in female but not male NPYCre/+;Y2^−/−^ mice were significantly increased (Figure 4C, 4D). Whole body fat mass measured by dual energy X-ray absorptiometry (DXA) showed a similar pattern of change to that of dissected fat mass in female mice (Figure 4E, 4G). Interestingly, there was an increase in whole body fat mass (as determined by DXA) in male Dox-injected NPYCre/+;Y2^−/−^ mice compared to saline- or Dox-injected wild type mice (Figure 4F), but when this effect was normalized for the effect of Dox *per se* there was no significant effect of Y2 receptor deletion on fat mass in male mice (Figure 4H). Lack of Y2 receptors on NPY expressing neurons does not influence whole body lean mass in either gender (Figure 4I, 4J).

**Figure 5:**
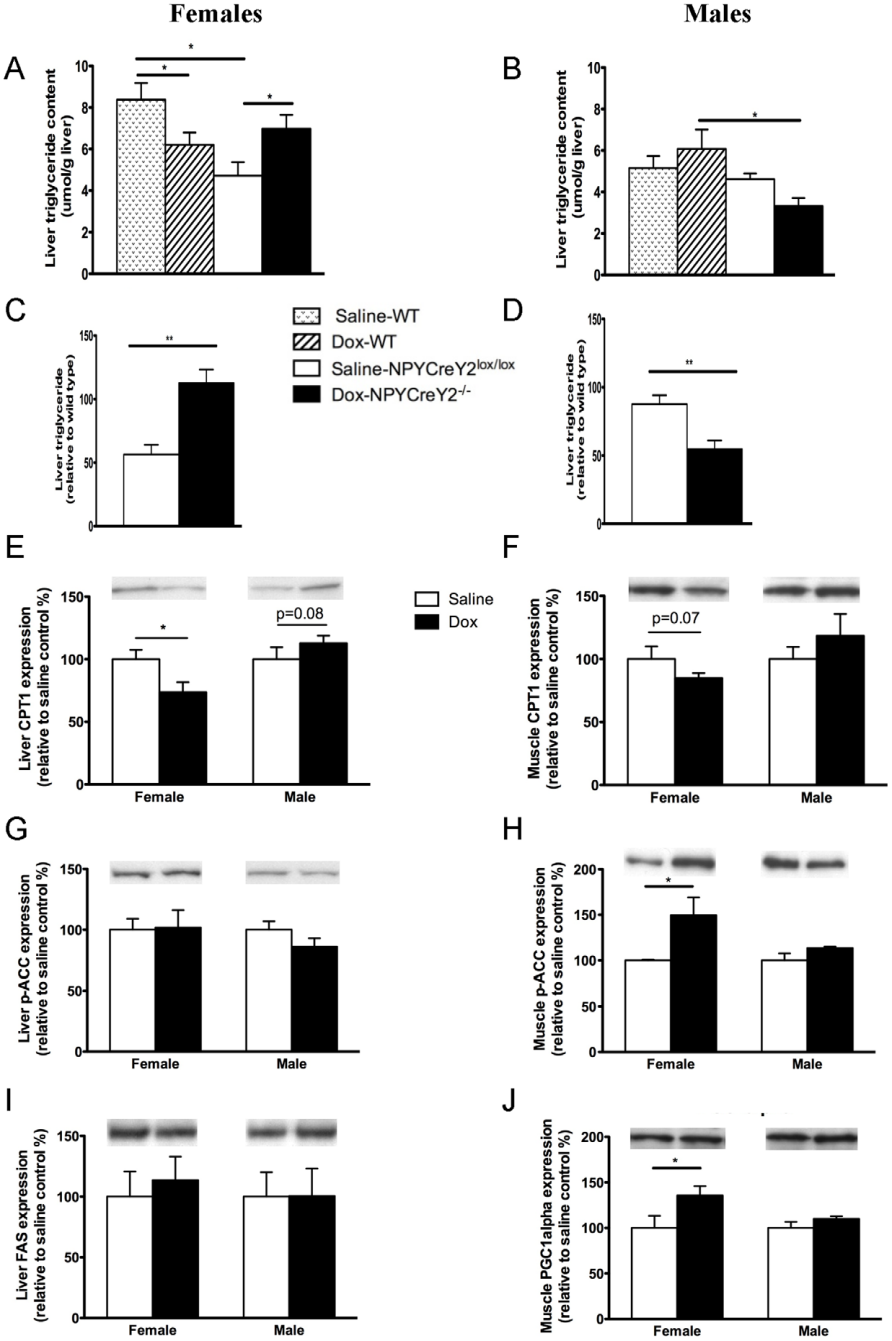
Effects of deleting Y2 receptors from hypothalamic NPY-expressing neurons on liver triglyceride content and expression of key proteins regulating energy metabolism in female and male NPYCre/+;Y2^lox/lox^ mice and wild type mice. (A, B) Liver triglyceride content in female and male wild type or NPYCre/+;Y2^lox/lox^ mice injected into the hypothalamus with either saline or doxycyline (Dox). (C, D) Liver triglyceride content of Dox-injected NPYCre/+;Y2^lox/lox^ mice relative to that of Dox-injected wild type mice (black columns) versus saline-injected NPYCre/+;Y2^lox/lox^ mice relative to saline-injected wild type mice (open columns). Protein levels (determined by Western blotting) of liver and muscle carmitoyl palmitate transferase 1 (CPT1) (E, F), liver and muscle phosphorylated acetyl-CoA carboxylase (p-ACC) (G, H), liver fatty acid synthase (FAS) (I) and muscle peroxisome proliferator-activated receptor (PPAR) γ coactivator (PGC1α) (J) in female or male NPYCre/+;Y2^lox/lox^ mice treated with saline or Dox. Data are means ± SEM of 6–12 mice per group. *, p<0.05 for the comparison indicated by horizontal bars.

In line with increased adiposity as determined by dissected white adipose tissue mass and DXA scans in females, we have also demonstrated that lack of Y2 receptors on NPY neurons increases hepatic triglyceride content in female mice (Figure 5A, 5C). This hepatic steatosis in females is associated with decreased protein expression (as determined by Western blotting) of carnitine palmitoyltransferase (CPT1), a key rate-limiting transmembrane enzyme controlling the entry of long chain fatty acid into mitochondria for oxidation, in both liver and skeletal muscle of female mice (Figure 5E, 5F). Moreover, the protein expression of phosphorylated acetyl-CoA carboxylase (p-ACC), a key enzyme in fatty acid synthesis, in the muscle but not liver was also up-regulated in these female mice (Figure 5G, 5H). Expression of protein for fatty acid synthase (FAS) in the liver was unchanged in female knockout mice (Figure 5I) but the muscle protein expression of peroxisome proliferator-activated receptor (PPAR) γ coactivator (PGC1α), a master regulator of oxidation in mitochondria, was significantly increased in the skeletal muscle of female NPYCre/+;Y2^−/−^ mice compared to that of controls (Figure 5J). Unlike the result in females, liver triglyceride content in male NPY neuron specific Y2 receptor deficient mice was markedly reduced (Figure 5B, 5D) in association with a trend towards increased liver and muscle CPT1 protein expression (Figure 5E, 5F) and no change in protein expression of liver or muscle phosphorylated ACC, FAS or PGC1α (Figure 5). Taken together, these findings suggest sex-specific involvement of Y2 receptors on NPY-expressing neurons in the regulation of energy metabolism, particularly adiposity, which could be mediated by changes in several key enzymes involved in lipolysis and lipogenesis in liver and skeletal muscle.

### Effect of NPY neuron specific Y2 receptor deletion on glucose metabolism

Some factors that regulate energy homeostasis do so in association with changes in glucose metabolism, even in the absence of effect on body weight or food intake [18]. We therefore investigated the effect of NPY neuron-specific Y2 receptor deletion on whole body glucose metabolism by examining glucose clearance during an intraperitoneal glucose tolerance test. Compared with control mice, female but not male mice with conditional NPY neuron-specific Y2 receptor deletion displayed improved fasting glycemia (Table 1). The areas under the glucose tolerance curves were not significantly different among groups (Table 1). These data indicate that Y2 receptors on NPY-ergic neurons are not directly involved in the regulation of glucose homeostasis.

### Effect of NPY neuron specific Y2 receptor deletion on bone homeostasis

**Figure 6:**
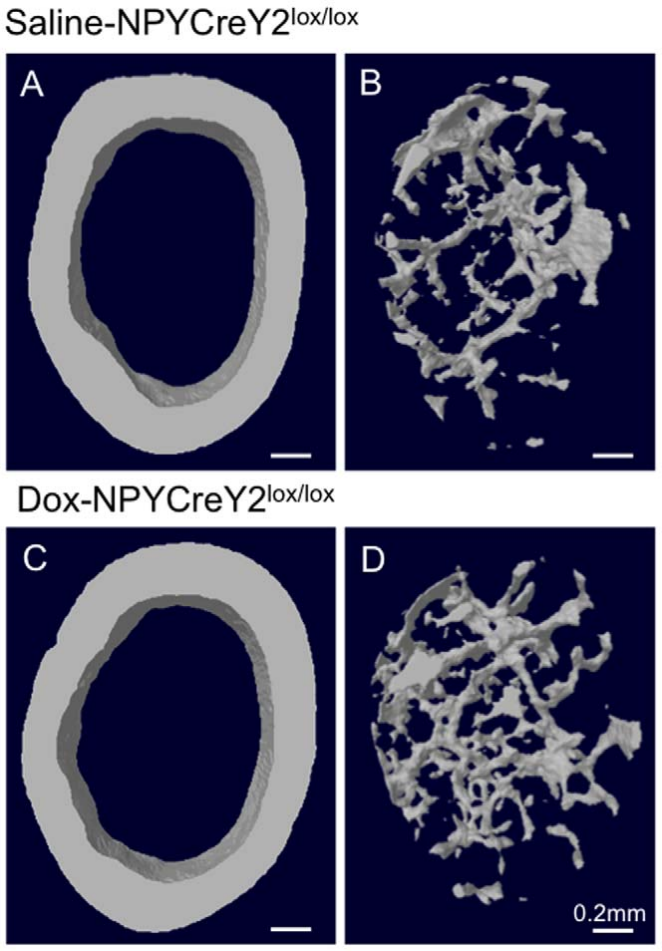
Effects of deleting Y2 receptors from hypothalamic NPY-expressing neurons on femoral morphology in saline-injected (control) versus Dox-injected (knockout) NPYCre/+;Y2^lox/lox^ mice. (A, C) Cross sectional microcomputed tomography (micro-CT) images of the cortical bone of representative male NPYCre/+;Y2^lox/lox^ mice injected into the hypothalamus with either saline or doxycyline (Dox). (B, D) cross-sectional micro-CT images of trabecular bone in the distal femora of male saline- or Dox-injected NPYCre/+;Y2^lox/lox^ mice. Images are representative of 6–8 male mice per group. Bar represents 0.2 mm.

We previously showed that germline Y2 receptor deletion leads to pronounced anabolic effects on cortical [30] as well as trabecular (cancellous) bone mass [31], and that adult-onset hypothalamus-specific Y2 receptor deletion leads to increased cortical bone mass [30] and significant increases in trabecular bone mass [31]. We therefore wanted to determine the contribution of Y2 receptors specifically expressed on NPY neurons to these processes. We thus measured whole body bone mineral density (BMD), whole body bone mineral content (BMC), isolated femur BMD and BMC and femur length in both genotypes and genders by dual energy X-ray absorptiometry (DXA, Table 3). There is no significant difference among groups in any of the above-mentioned parameters. As cortical bone is the predominant type of bone detected by DXA scans of the whole body or of isolated femora, these data indicate that Y2 receptors on non-NPY expressing neurons in the hypothalamus do not regulate cortical bone homeostasis. This finding is corroborated by the detailed structural analysis by microcomputed tomography (micro-CT), which revealed no difference between genotypes in the volume or thickness of cortical bone in the distal femora (0.91±0.02 mm^3^ and 0.22±0.005 mm in Dox- versus 0.91±0.02 mm^3^ and 0.22±0.004 mm in saline-injected NPYCre/+;Y2^lox/lox^ (data are means±SEM of 6–8 male mice per group). Representative micro-CT scans of cortical bone are shown in Figure 6A and 6C. In contrast to this lack of effect on cortical bone, Y2 receptor deletion on hypothalamic NPY-expressing neurons resulted in slight (1.2-fold) but significant increases in trabecular bone volume and trabecular number as determined by analysis of micro-CT scans (6.72±0.15% of total tissue volume and 1.32±0.02 trabeculae per mm in Dox- versus 5.58±0.46% and 1.09±0.09 trabeculae per mm in saline-injected NPYCre/+;Y2^lox/lox^ (data are means ± SEM of 6–8 male mice per group, p = 0.02 for both comparisons), with no change in trabecular thickness (data not shown). These findings are illustrated by the representative micro-CT scans in figures 6B and 6D. This results indicates that Y2 receptors on NPY-expressing neurons in the hypothalamus influence trabecular bone, albeit other Y2 receptors probably contribute to the pronounced 2-fold increase in trabecular bone volume that was observed in mice in which Y2 receptors were deleted in the hypothalamus [31].

**Table 3:** Effects of NPY neuron-specific Y2 receptor deletion on bone parameters determined by dual energy X-ray absorptiometry (DXA).

Parameters	Female				Male			
	WT		NPYCre/+;Y2^lox/lox^		WT		NPYCre/+;Y2^lox/lox^	
	Saline (5)	Dox (5)	Saline (7)	Dox (8)	Saline (5)	Dox (7)	Saline (6)	Dox (12)
**Whole body BMD (g/mm^3^)**	0.049±0.002	0.05±0.003	0.051±0.001	0.052±0.001	0.052±0.001	0.053±0.001	0.053±0.001	0.054±0.001
**Isolated femur BMD (g/mm^3^)**	0.056±0.002	0.059±0.002	0.058±0.002	0.06±0.001	0.057±0.002	0.059±0.002	0.056±0.001	0.057±0.001
**Whole body BMC (g)**	0.313±0.029	0.324±0.033	0.334±0.021	0.354±0.012	0.372±0.022	0.394±0.012	0.369±0.007	0.386±0.009
**Isolated femur BMC (g)**	0.019±0.001	0.020±0.001	0.021±0.001	0.022±0.001	0.024±0.001	0.024±0.001	0.024±0.001	0.025±0.001
**Femur length (mm)**	15.76±0.36	15.98±0.18	15.91±0.20	15.87±0.19	16.57±0.19	16.66±0.13	16.14±0.18	16.14±0.08

Whole body bone mineral density (BMD), bone mineral content (BMC) and isolated femur BMD and BMC were measured by DXA. Values are means ± SEM of 5–12 mice per group as shown in parentheses.

## Discussion

Y2 receptor signalling has been shown to be critical for the regulation of both adipose tissue and bone homeostasis, with germline knockout of Y2 receptors reducing adiposity and increasing bone formation [26], [27], [30], [31]. In the present study we now demonstrate that hypothalamic Y2 receptor deletion in adult mice results in sustained positive energy balance characterized by hyperphagia, increased body weight gain and a marked trend towards increased fat accretion in white adipose tissue. In this study we also generated a unique conditional NPY neuron-specific Y2 receptor knockout model, which allows the first *in vivo* studies of the within cell relationship between NPY production and Y2 auto-receptor signalling, and their subsequent effect on energy balance and bone homeostasis. We thus demonstrated that adult-onset selective deletion of Y2 receptors from hypothalamic NPY-expressing neurons leads to significant female-specific increases in adiposity and hepatic steatosis in the absence of changes in spontaneous or fasting-induced food intake or body weight, as well as increased body weight in male and female mice under conditions of stress induced by a new environment. Importantly, we also showed that specific lack of Y2 receptor signalling on hypothalamic NPY-expressing neurons leads to a slight but significant increase in trabecular bone volume and trabecular number, but is not critical for the regulation of glucose metabolism or cortical bone homeostasis, suggesting a differential role of Y2 receptors in the regulation of energy homeostasis and cortical bone mass that is dependent on location.

Lack of Y2 receptors may cause net anabolic effects via removal of presynaptic inhibition of NPY synthesis and inhibition on POMC-expressing neurons. Consistent with this hypothesis, deletion of Y2 receptors on hypothalamic NPY-ergic neurons results in a significant increase in NPY mRNA levels in the Arc compared to saline-injected control mice. For the first time, this result provides direct *in vivo* evidence of a negative feedback mechanism between NPY expression and Y2 auto-receptors. Interestingly, besides an increase in NPY mRNA levels, POMC mRNA levels are markedly down regulated in the Arc of mice lacking Y2 receptors on NPY-expressing neurons. It is likely that increased activity of NPY-ergic neurons enhances the inhibitory effect of GABA-ergic neurotransmission to POMC neurons, thereby decreasing POMC mRNA levels in the Arc [27], [41]. This hypothalamic profile of increased NPY and decreased POMC expression is consistent with that seen in germline Y2 receptor knockout mice, but is different from the overall hypothalamic Y2 receptor deletion model in which POMC mRNA levels are increased, likely due to secondary adaptations [26]. These findings suggest a catabolic role of Y2 receptors on NPY-ergic neurons, and loss of these receptors in adult mice releases the inhibition on NPY activity thereby leading to anabolic effects. Furthermore, these data indicate that Y2 receptors on NPY neurons are indirectly involved in the regulation of POMC mRNA levels in the Arc, thereby exerting a further influence on energy balance.

This model of hypothalamic NPY neuron-selective Y2 receptor deletion demonstrates increased white adipose tissue mass and hepatic steatosis in female but not in male mice. There are a few possible explanations for these findings. For instance, lack of PYY-induced satiety [42] and PYY-induced catabolic effects [43] acting via hypothalamic Y2 receptors may contribute to the elevated adiposity [42] in female mice. Moreover, increased hypothalamic NPY mRNA and decreased POMC mRNA may, at least in part, contribute to a peripheral increase in adiposity and liver triglyceride content in females. Previous studies have shown that female rodents are more responsive than males to the obesogenic effects of centrally-administered NPY [19]. In keeping with this, germline and hypothalamus-specific Y2 receptor knockout also led to differential effects on food intake, adiposity, serum pancreatic peptide and corticosterone concentrations in male and female mice [26]. It is likely that there exists an interaction between Y2 receptor signalling and sex steroid pathways in the regulation of several processes involved in energy homeostasis. Indeed, germline deletion of Y2 receptors prevents the elevation in WAT mass following ovariectomy, and Y2 receptors other than those located in the hypothalamus are most likely responsible for this effect [44]. Additionally, imbalances between lipolytic and lipogenic pathways in the liver and skeletal muscles, two major metabolically active tissues involved in energy homeostasis, may be underpinning the sex-specific changes in triglyceride accumulation in these animals. For example, CPT1 is the rate-limiting transmembrane enzyme for the entry of long-chain fatty acids into mitochondria for β-oxidation. The decreased expression of CPT1 in female mice (and the trend towards increased expression of CPT1 in males) highlights the sex-specific role of Y2 receptors on NPY neurons in the control of fatty acid influx into mitochondria for oxidation in the liver and skeletal muscle. Additionally, biosynthesis of fatty acid is likely, at least in part, to also be regulated by NPY neuron specific Y2 signalling, as evident by altered expression levels of acetyl-CoA carboxylase (ACC) in skeletal muscle of female mice, which could subsequently inversely influence expression of CPT1.

Despite the significant alterations in fat mass and hepatic triglyceride levels in female hypothalamic NPY neuron-specific Y2 deficient mice, feeding behaviour and body weight were not altered in male or female knockouts, although significantly increased NPY and decreased POMC mRNA levels in the Arc would have been predicted to induce hyperphagia and weight gain. However, changes in adipose tissue depot weights are not always associated with commensurate changes in body weight or food intake in experimental animals [45], [46]. Moreover, the Y2 receptor deletion-induced increase in hypothalamic NPY expression levels may not necessarily induce feeding responses, consistent with findings from NPY knockout mice [16]. The orexigenic effect of NPY is mediated via other receptors besides Y2, notably Y1 receptors, because hyperphagia is significantly reduced in Y1 receptor-deficient *ob/ob* mice [47]. Importantly, studies have shown that a proportion of Y2 receptors are expressed on non-NPY containing neurons in the Arc [28], and therefore the lack of effect of NPY neuron-specific Y2 receptor deletion on feeding may be due to the complex multiple effects of Y2 receptors.

Interestingly, the anti-obesogenic effects of germline Y2 receptor knockout [26], [27], [48] were opposite to the lipogenic effects of hypothalamus-specific Y2 receptor knockout or hypothalamic NPY neuron-specific Y2 deletion demonstrated in this study. This finding indicates mediation of an anti-obesogenic effect by Y2 receptors other than those hypothalamic Y2 receptors that were deleted in the present study. Selective Y2 receptor deletion in peripheral tissues would enable us to understand the role of peripheral Y2 receptors in the regulation of bone and adipose tissue. The wide expression of Y2 receptors in peripheral tissues, including adipose tissue and the pancreas [49], highlights the potential role of peripheral Y2 signalling in the regulation of energy homeostasis.

Deletion of Y2 receptors specifically in the basolateral and central amygdala induces anxiolytic effects [50]. Although our Y2 receptor knockout strategy targeted Y2 receptors on NPY-expressing neurons in the hypothalamus, we cannot exclude the possibility that other populations of Y2 receptors on NPY-expressing neurons (such as in the amygdala) were also deleted, which could have contributed to reduced stress responses and thus the increased adiposity observed in female mice lacking Y2 receptors on NPY-expressing neurons, and also to the increased body weight observed in both female and male knockouts in the novel environment of indirect calorimetry cages, since novel stressors are known to induce weight loss in rodents [27], [51], [52], [53]. Y2 receptors are known to regulate stress responses as well as activity of the hypothalamo-pituitary adrenal axis, with germline Y2 receptor ablation reducing anxiety [49], [54], [55], [56] and decreasing hypothalamic expression of corticotropin releasing hormone and the circulating concentrations of corticosterone in lean or obese (*ob/ob* or diet-induced obese) mice [27], [57]. The present findings suggest that NPY neuron-specific Y2 receptors in the brain may mediate these effects of germline Y2 receptor ablation, and further studies dedicated to elucidating stress responses in NPYCre/+;Y2^−/−^ mice in the regulation of energy balance are warranted.

Importantly, our data reveal that hypothalamic NPY neuron specific Y2 receptor knockout has significant effects on trabecular bone homeostasis, as evident from significant increases in trabecular bone volume and trabecular number in femora from knockout versus control mice. Because the magnitude of these changes was not as great as the marked increase in these parameters observed in response to overall Y2 receptor knockout in the hypothalamus of adult mice [31], Y2 receptors on non-NPY-ergic neurons must play a key role in the hypothalamic regulation of trabecular bone homeostasis. In contrast, we saw that Y2 ablation on NPY-expressing neurons in the hypothalamus has no effect on cortical bone homeostasis, as indicated by unaltered bone mineral density or bone mineral content in these conditional Y2 knockout mice of either gender and no change in cortical volume or thickness. This is in contrast to the increased cortical bone mass observed in germline and hypothalamus-specific Y2 receptor deficient mice [30], [31]. One possibility for this discrepancy could be that parallel Y2 receptor-activated pathways in the hypothalamus are responsible for cortical bone regulatory functions, and effects of lack of Y2 receptors only in NPY-ergic neurons might be compensated for by another Y2 receptor pathway. Taken together, these findings highlight the importance of specifically investigating the role of post-synaptic Y2 receptors on non-NPY neurons in the regulation of bone mass and energy metabolism.

**Figure 7:**
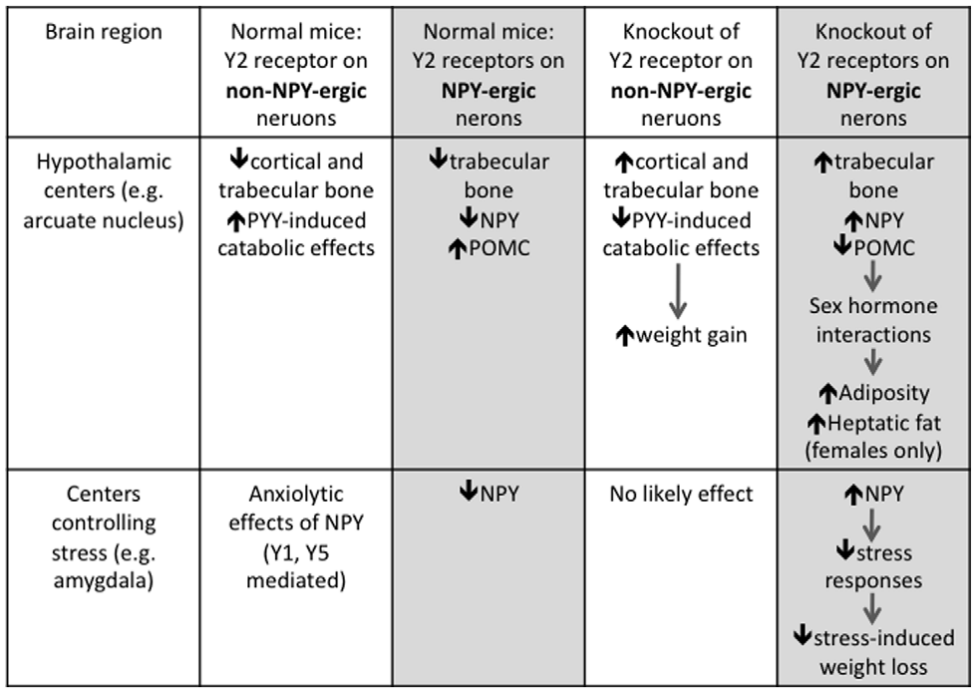
Summary of the effects of deletion of Y2 receptors on non-NPY-ergic or NPY-ergic neurons in the brain. In normal mice, Y2 receptors on non-NPY-ergic neurons in the hypothalamus (e.g. in the arcuate nucleus) reduce cortical and trabecular bone mass, as evidenced by the fact that hypothalamic ablation of Y2 receptors on non-NPY-ergic as well as on NPY-ergic neurons increases cortical and trabecular bone mass [31], whereas ablation of Y2 receptors specifically from NPY-ergic neurons in the current study only partially increases trabecular bone mass and has no effect on cortical bone mass. Additionally in normal mice, Y2 receptors on non-NPY-ergic neurons in brain centres regulating stress responses (such as the amygdala) are not likely involved in mediating the anxiolytic effects of NPY, which are likely mediated by Y1 and Y5 receptors [56]. However, Y2 receptors on NPY-ergic neurons in the amygdala induce anxiogenic effects [50], likely due to decreased endogenous NPY-ergic action [56]. Our current work shows that when Y2 receptors are deleted specifically from NPY-ergic neurons in the brain, there is a resultant increase in NPY and a decrease in POMC mRNA expression levels in the arcuate nucleus of the hypothalamus, and this – in conjunction with likely sex hormone interactions – could contribute to the increased adiposity and hepatic fat content observed in these female knockouts. In addition to effects mediated by the hypothalamus, Y2 receptor deletion on NPY-ergic neurons in centres controlling stress responses may increase NPY-ergic actions in brain regions such as the amygdala, which can thereby reduce stress responses [50], [56] and probably contribute to the absence of stress-induced weight loss in male and female mice lacking Y2 receptors specifically from NPY-ergic neurons. An additional mechanism by which central Y2 deletion can promote positive energy balance could be via blockade of PYY-induced catabolic effects [43] on non-NPY-ergic or NPY-ergic neurons or both.

In summary, we generated an adult-onset conditional NPY neuron-specific Y2 receptor knockout model that has enabled us to investigate the role of Y2 receptors specifically on hypothalamic NPY-expressing neurons in the control of adipose tissue and bone homeostasis. This study revealed no direct role of this type of Y2 receptor in cortical skeletal regulation, and it also provides clear evidence of sex-specific functions in the regulation of trabecular bone mass, adiposity and hepatic triglyceride accumulation, and that these effects are achieved in association with changes in the balance between lipogenic signalling pathways and lipolytic signalling pathways in the liver and skeletal muscle, as summarized in Figure 7. More importantly, this study has shown for the first time the direct relationship between NPY production and Y2 auto-receptors in the Arc *in vivo* (Figure 7). The results of this study suggest that enhancing the catabolic effect of NPY-ergic Y2 receptors may open up potential new ways to treat obesity without any adverse effects on cortical bone mass, the major determinant of bone strength.

## References

[1] Stanley BG, Anderson KC, Grayson MH, Leibowitz SF (1989). Repeated hypothalamic stimulation with neuropeptide Y increases daily carbohydrate and fat intake and body weight gain in female rats.. Physiol Behav.

[2] Kotz CM, Briggs JE, Grace MK, Levine AS, Billington CJ (1998). Divergence of the feeding and thermogenic pathways influenced by NPY in the hypothalamic PVN of the rat.. Am J Physiol.

[3] Baraban SC (1998). Neuropeptide Y and limbic seizures.. Reviews in the Neurosciences.

[4] Lin S, Boey D, Herzog H (2004). NPY and Y receptors: lessons from transgenic and knockout models.. Neuropeptides.

[5] Batterham RL, Cowley MA, Small CJ, Herzog H, Cohen MA (2002). Gut hormone PYY_3-36_ physiologically inhibits food intake.. Nature.

[6] Dowell P, Hu Z, Lane M (2005). Monitoring energy balance: metabolites of fatty acid synthesis as hypothalamic sensors.. Annual Review of Biochemistry.

[7] Hahn TM, Breininger JF, Baskin DG, Schwartz MW (1998). Coexpression of Agrp and NPY in fasting-activated hypothalamic neurons.. Nat Neurosci.

[8] Ellacott KL, Cone RD (2004). The central melanocortin system and the integration of short- and long-term regulators of energy homeostasis.. Recent Progres in Hormone Research.

[9] Blomqvist AG, Herzog H (1997). Y-receptor subtypes–how many more?. Trends Neurosci.

[10] Ollmann MM, Wilson BD, Yang YK, Kerns JA, Chen Y (1997). Antagonism of central melanocortin receptors in vitro and in vivo by agouti-related protein.. Science.

[11] Acuna-Goycolea C, Tamamaki N, Yanagawa Y, Obata K, van den Pol AN (2005). Mechanisms of Neuropeptide Y, Peptide YY, and Pancreatic Polypeptide inhibition of identified green fluorescent protein-expressing GABA neurons in the hypothalamic neuroendocrine arcuate nucleus.. The Journal of Neuroscience.

[12] Beck B, Burlet A, Bazin R, Nicholas JP, Burlet C (1993). Elevated neuropeptide Y in the arcuate nucleus of young obese Zucker rats may contribute to the development of their overeating.. Journal of Nutrition.

[13] Kalra SP, Dube MG, Sahu A, Phelps CP, Kalra PS (1991). Neuropeptide Y secretion increases in the paraventricular nucleus in association with increased appetite for food.. Proceedings of the National Academy of Sciences of the United States of America.

[14] McKibbon PE, Cotton SJ, McMillan S, Holloway B, Mayers R (1991). Altered neuropeptide Y concentrations in specific hypothalamic regions of obese (fa/fa) Zucker rats. Possible relationship to obesity and neuroendocrine disturbances.. Diabetes.

[15] Williams G, Steel JH, Cardoso H, Ghatei MA, Lee YC (1988). Increased hypothalamic neuropeptide Y concentrations in diabetic rat.. Diabetes.

[16] Erickson JC, Clegg KE, Palmiter RD (1996). Sensitivity to leptin and susceptibility to seizures of mice lacking neuropeptide Y.. Nature.

[17] Erickson JC, Hollopeter G, Palmiter RD (1996). Attenuation of the obesity syndrome of ob/ob mice by the loss of neuropeptide Y.. Science.

[18] Sainsbury A, Cusin I, Rohner-Jeanrenaud F, Jeanrenaud B (1997). Adrenalectomy prevents the obesity syndrome produced by chronic central neuropeptide Y infusion in normal rats.. Diabetes.

[19] Zarjevski N, Cusin I, Vettor R, Rohner-Jeanrenaud F, Jeanrenaud B (1993). Chronic intracerebroventricular neuropeptide-Y administration to normal rats mimics hormonal and metabolic changes of obesity.. Endocrinology.

[20] Pierroz DD, Catzeflis C, Aebi AC, Rivier JE, Aubert ML (1996). Chronic administration of neuropeptide Y into the lateral ventricle inhibits both the pituitary-testicular axis and growth hormone and insulin-like growth factor I secretion in intact adult male rats.. Endocrinology.

[21] Billington CJ, Briggs JE, Harker S, Grace M, Levine AS (1994). Neuropeptide Y in hypothalamic paraventricular nucleus: a center coordinating energy metabolism.. Am J Physiol.

[22] Parker RMC, Herzog H (1999). Regional distribution of Y-receptor subtype mRNAs in rat brain.. European Journal of Neuroscience.

[23] Fetissov SO, Byrne LC, Hassani H, Ernfors P, Hokfelt T (2004). Characterization of neuropeptide Y Y2 and Y5 receptor expression in the mouse hypothalamus.. Journal of Comparative Neurology.

[24] Leibowitz SF, Alexander JT (1991). Analysis of neuropeptide Y-induced feeding: dissociation of Y1 and Y2 receptor effects on natural meal patterns.. Peptides.

[25] King PJ, Williams G, Doods H, Widdowson PS (2000). Effect of a selective neuropeptide Y Y(2) receptor antagonist, BIIE0246 on neuropeptide Y release.. Eur J Pharmacol.

[26] Sainsbury A, Schwarzer C, Couzens M, Fetissov S, Furtinger S (2002). Important role of hypothalamic Y2 receptors in body weight regulation revealed in conditional knockout mice.. Proc Natl Acad Sci U S A.

[27] Sainsbury A, Schwarzer C, Couzens M, Herzog H (2002). Y2 receptor deletion attenuates the type 2 diabetic syndrome of ob/ob mice.. Diabetes.

[28] Broberger C, Landry M, Wong H, Walsh JN, Hokfelt T (1997). Subtypes Y1 and Y2 of the neuropeptide Y receptor are respectively expressed in pro-opiomelanocortin- and neuropeptide-Y-containing neurons of the rat hypothalamic arcuate nucleus.. Neuroendocrinology.

[29] Baldock PA, Sainsbury A, Allison S, Lin EJ, Couzens M (2005). Hypothalamic control of bone formation: distinct actions of leptin and Y2 receptor pathways.. Journal of Bone and Mineral Research.

[30] Baldock PA, Allison S, McDonald MM, Sainsbury A, Enriquez RF (2006). Hypothalamic regulation of cortical bone mass: opposing activity of Y2 receptor and leptin pathways.. J Bone Miner Res.

[31] Baldock PA, Sainsbury A, Couzens M, Enriquez RF, Thomas GP (2002). Hypothalamic Y2 receptors regulate bone formation.. J Clin Invest.

[32] Baldock PA, Lee NJ, Driessler F, Lin S, Allison S (2009). Neuropeptide Y knockout mice reveal a central role of NPY in the coordination of bone mass to body weight.. PLoS One.

[33] Sainsbury A, Baldock P, Schwarzer C, Ueno H, Enriquez R (2003). Synergistic effects of Y2 and Y4 receptors on adiposity and bone mass revealed in double knockout mice.. Molecular and Cellular Biology.

[34] Lin S, Lin EJ, Boey D, Lee NJ, Slack K (2007). Fasting inhibits the growth and reproductive axes via distinct Y2 and Y4 receptor-mediated pathways.. Endocrinology.

[35] Franklin KBP, Paxinos G (1997). The Mouse Brain in Stereotaxic Coordinates..

[36] Ferrannini E (1988). The theoretical bases of indirect calorimetry: a review.. Metabolism.

[37] Frayn KN (1983). Calculation of substrate oxidation rates in vivo from gaseous exchange.. J Appl Physiol.

[38] McLean JA, Tobin G (1987). Animal and human Calorimetry.

[39] Ye JM, Iglesias MA, Watson DG, Ellis B, Wood L (2003). PPARalpha/gamma ragaglitazar eliminates fatty liver and enhances insulin action in fat-fed rats in the absence of hepatomegaly.. Am J Physiol Endocrinol Metab.

[40] Gossen M, Freundlieb S, Bender G, Muller G, Hillen W (1995). Transcriptional activation by tetracyclines in mammalian cells.. Science.

[41] Cowley MA, Smart JL, Rubinstein M, Cerdan MG, Diano S (2001). Leptin activates anorexigenic POMC neurons through a neural network in the arcuate nucleus.. Nature.

[42] Boey D, Lin S, Karl T, Baldock P, Lee N (2006). Peptide YY ablation in mice leads to the development of hyperinsulinemia and obesity.. Diabetologia.

[43] Boey D, Lin S, Enriquez RF, Lee NJ, Slack K (2008). PYY transgenic mice are protected against diet-induced and genetic obesity.. Neuropeptides.

[44] Allison SJ, Baldock P, Sainsbury A, Enriquez R, Lee NJ (2006). Conditional deletion of hypothalamic Y2 receptors reverts gonadectomy-induced bone loss in adult mice.. J Biol Chem.

[45] Baran K, Preston E, Wilks D, Cooney GJ, Kraegen EW (2002). Chronic central melanocortin-4 receptor antagonism and central neuropeptide-Y infusion in rats produce increased adiposity by divergent pathways.. Diabetes.

[46] Pedrazzini T, Seydoux J, Kunstner P, Aubert JF, Grouzmann E (1998). Cardiovascular response, feeding behavior and locomotor activity in mice lacking the NPY Y1 receptor.. Nat Med.

[47] Pralong FP, Gonzales C, Voirol MJ, Palmiter RD, Brunner HR (2002). The neuropeptide Y Y1 receptor regulates leptin-mediated control of energy homeostasis and reproductive functions.. Faseb J.

[48] Naveilhan P, Svensson L, Nystrom S, Ekstrand AJ, Ernfors P (2002). Attenuation of hypercholesterolemia and hyperglycemia in ob/ob mice by NPY Y2 receptor ablation.. Peptides.

[49] Playford RJ, Cox HM (1996). Peptide YY and neuropeptide Y: two peptides intimately involved in electrolyte homeostasis.. Trends Pharmacol Sci.

[50] Tasan RO, Nguyen NK, Weger S, Sartori SB, Singewald N (2010). The central and basolateral amygdala are critical sites of neuropeptide Y/Y2 receptor-mediated regulation of anxiety and depression.. J Neurosci.

[51] Marin MT, Cruz FC, Planeta CS (2007). Chronic restraint or variable stresses differently affect the behavior, corticosterone secretion and body weight in rats.. Physiol Behav.

[52] Zafar HM, Pare WP, Tejani-Butt SM (1997). Effect of acute or repeated stress on behavior and brain norepinephrine system in Wistar-Kyoto (WKY) rats.. Brain Res Bull.

[53] Kuo LE, Kitlinska JB, Tilan JU, Li L, Baker SB (2007). Neuropeptide Y acts directly in the periphery on fat tissue and mediates stress-induced obesity and metabolic syndrome.. Nat Med.

[54] Redrobe JP, Dumont Y, Herzog H, Quirion R (2004). Characterization of neuropeptide Y, Y(2) receptor knockout mice in two animal models of learning and memory processing.. J Mol Neurosci.

[55] Tschenett A, Singewald N, Carli M, Balducci C, Salchner P (2003). Reduced anxiety and improved stress coping ability in mice lacking NPY-Y2 receptors.. Eur J Neurosci.

[56] Heilig M (2004). The NPY system in stress, anxiety and depression.. Neuropeptides.

[57] Sainsbury A, Bergen HT, Boey D, Bamming D, Cooney GJ (2006). Y2Y4 receptor double knockout protects against obesity due to a high-fat diet or Y1 receptor deficiency in mice.. Diabetes.

